# Modified Laparoscopic Sugarbaker Repair of Parastomal Hernia With a Totally Extraperitoneal Technique

**DOI:** 10.3389/fsurg.2021.740430

**Published:** 2021-10-05

**Authors:** Huiyong Jiang, Dil Momin Thapa, Xiangjun Cai, Chun Ma, Mofei Wang

**Affiliations:** ^1^The Second Department of General Surgery, Northeast International Hospital, Shenyang, China; ^2^Clinical Medical School, Inner Mongolia University for the Nationalities, Tongliao, China; ^3^The Second Department of General Surgery, The Affiliated Hospital of Inner Mongolia University for the Nationalities, Tongliao, China

**Keywords:** laparoscopic, extraperitoneal, parastomal hernia, mesh, sugarbaker

## Abstract

**Purpose:** Many patients develop a parastomal hernia within the first 2 years of stoma formation, and even surgical repair is associated with high recurrence rates. An intraperitoneal approach is typically used for the laparoscopic repair of parastomal hernia; it is unknown whether a totally extraperitoneal technique (TEP) is feasible. Here we describe a laparoscopic TEP approach using a modified Sugarbaker method for the repair of parastomal hernia.

**Methods:** Seven patients underwent parastomal hernia repair. The retrograde puncture technique was used to create the extrapneumoperitoneum, and the peritoneum was separated with a laparoscopic TEP approach; the mesh was placed using a modified Sugarbaker technique.

**Results:** All patients had an oncologic etiology for stoma creation. The mean (±SD) size of the hernia defect was 3.1 ± 2.7 cm and the mesh size was 303.4 ± 96.8 cm^2^. The mean operative time was 195.5 ± 20.7 min and average length of hospital stay after surgery was 4.8 ± 2.1 days. One patient had intraoperative subcutaneous emphysema. The average follow-up time was 8.5 ± 2.7 months; mild pain occurred in 2 patients, 3 experienced seroma formation (with no special treatment required), and 1 had early intestinal obstruction (which was treated with conservative care). There was no hernia recurrence, wound complications, or infections of the surgical site or mesh during follow-up.

**Conclusion:** A laparoscopic TEP technique is technically challenging but feasible. Modified laparoscopic Sugarbaker repair of a parastomal hernia with the TEP technique is safe and effective, although the recurrence rate and late complications require confirmation in more cases with long-term follow-up.

## Introduction

Parastomal hernias are the most common long-term complication following ostomy surgery, with a very high incidence rate (50%) and recurrence rate even after repair (18%) (1, 2). Various techniques such as open, laparoscopic, and robotic surgery have been described for parastomal hernia repair (3). Commonly used laparoscopic repair techniques are the keyhole, Sugarbaker, and sandwich (4–6). An intraperitoneal approach is typically used for the laparoscopic repair of parastomal hernia, which involves placing an anti-adhesion mesh with a special coating in contact with the bowel. However, mesh placement within the peritoneum carries the risk of intestinal adhesion or fistula, which can be life-threatening (7).

Satisfactory results have been achieved in the laparoscopic repair of various types of hernia using the totally extraperitoneal (TEP) technique (8–12). However, the laparoscopic repair of parastomal hernia with a TEP technique rarely been reported, and there is little evidence of laparoscopic application. We previously reported the successful separation of the peritoneum with this technique and repair of a parastomal hernia using a synthetic mesh without anti-adhesion coating (12). In that case, the sigmoid colon was short and adhered to the ventral wall with a certain degree of tension; we therefore selected a keyhole-like method for laparoscopy (12). However, repair of a parastomal hernia using a modified laparoscopic Sugarbaker method is preferable to a keyhole mesh (1) because the bowel at the stoma can be treated in the same manner as the spermatic cord in inguinal hernia surgery. Here we describe laparoscopic parastomal hernia repair in 7 patients using a TEP approach and Sugarbaker mesh configuration. This technique eliminates the need to move the stoma or cut the mesh during its placement.

## Surgical Technique

A standard surgical technique was used for all patients. General anesthesia was used in all surgeries, with the patients placed in the supine and Trendelenburg position. The patient's abdomen was painted with 7.5% povidone–iodine and a surgical scrub was placed from the nipple to the knee. The peristomal area was cleaned with 10% povidone–iodine solution and the stoma was sealed with an adhesive incise drape. The surgeons were standing contralateral to the stoma with the monitor over the surgical site.

### Step 1

**Figure 1:**
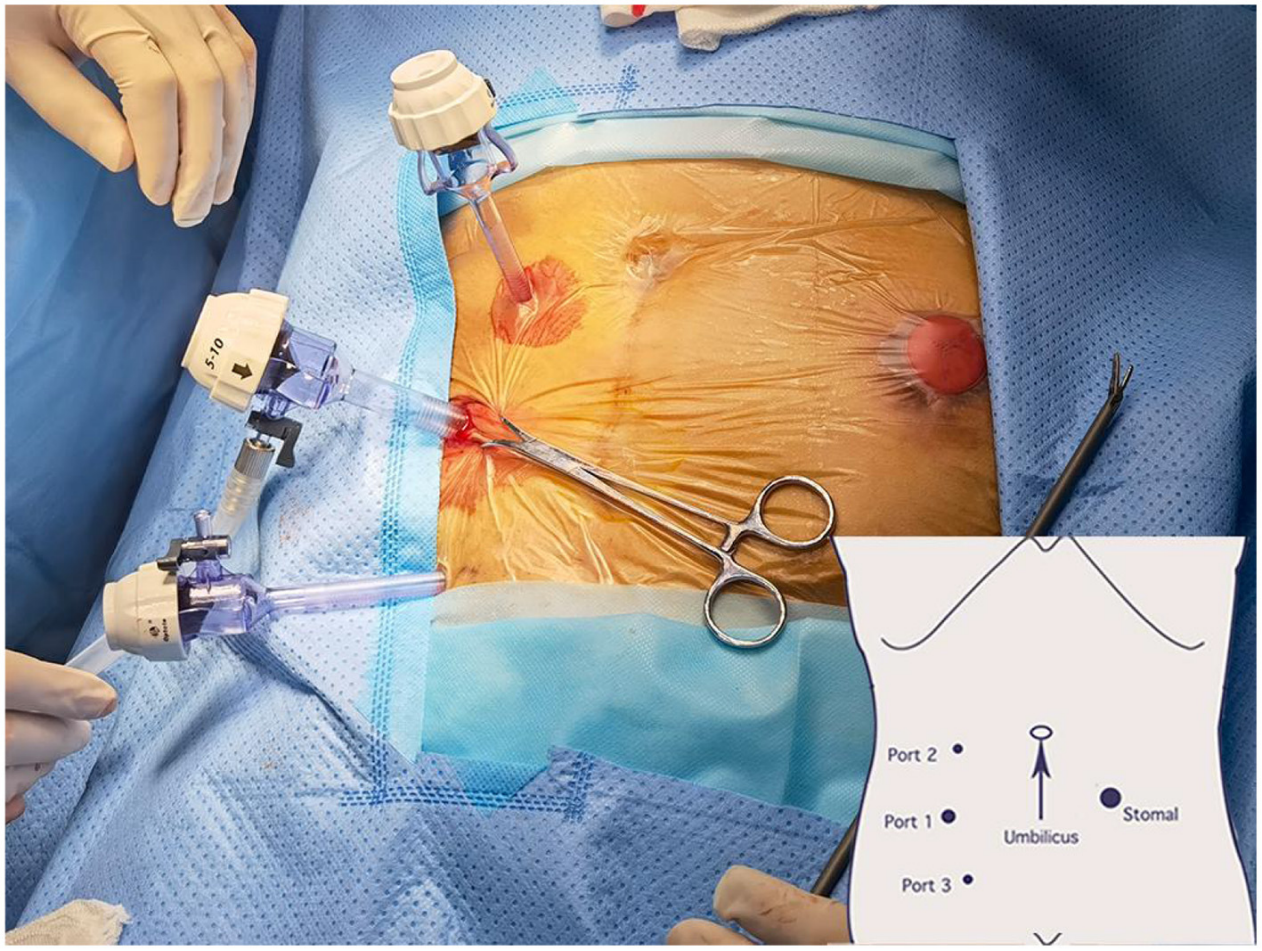
Trocar placement.

A 1.2 cm incision was made through the skin and subcutaneous adipose tissue in the right iliac region. Retractors were used to separate and expose the incised subcutaneous tissue and further dissect the aponeurosis of the external oblique muscle. Hemostatic forceps were used for separation of the internal oblique and transversus abdominis muscles. The primary extraperitoneal space was created using an index finger. A total of 3 trocars (2 × 5 and 1 × 12 mm) were used for this procedure. The trocar placement is shown in Figure 1 and has been previously described (12, 13). After successfully placing all 3 trocars, an extrapneumoperitoneum was created using CO_2_ (11 mmHg CO_2_) for laparoscope insertion.

### Step 2

**Figure 2:**
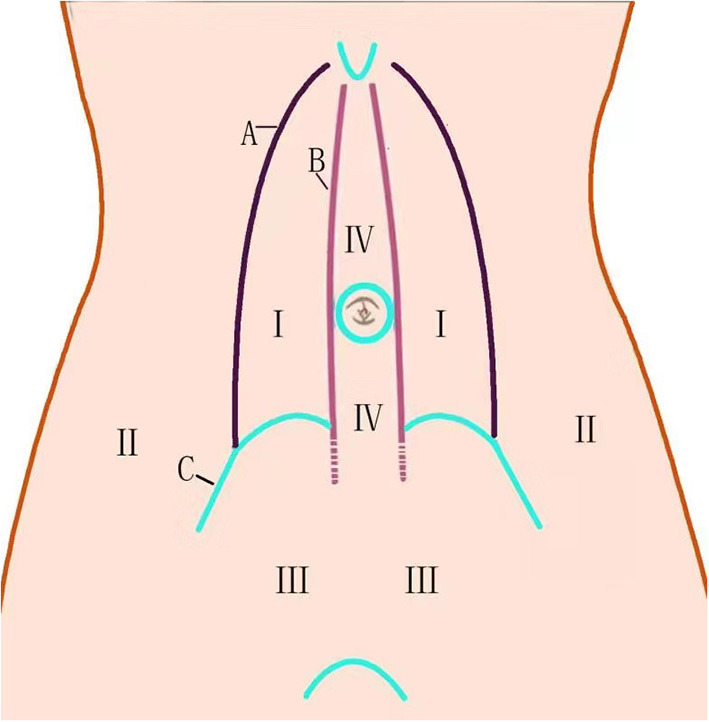
Extraperitoneal space in abdominal wall.

**Figure 3:**
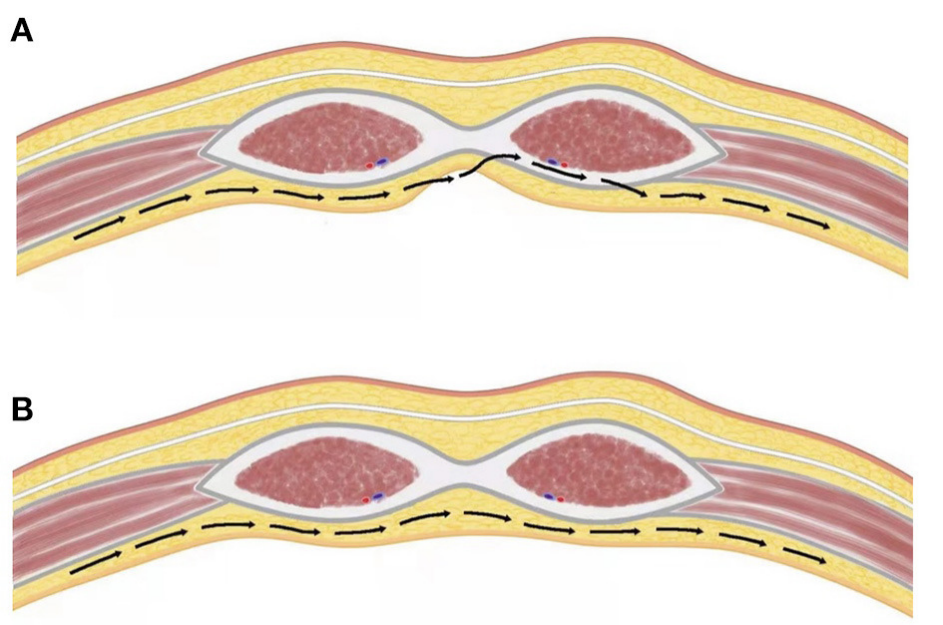
Route of extraperitoneal approach. **(A)** Incision in the peritoneum. **(B)** Totally laparoscopic approach.

Under direct laparoscopic guidance, a larger extraperitoneal space was created down to the pubic floor and up to the bladder (Figure 2-III), where it was easier to create. Space was then created to the contralateral side of the abdomen (Figure 2-I, IV). The long midline incision in the abdomen made it very difficult to separate the peritoneum; in cases where adhesiolysis was challenging, the membrane was cut while avoiding damage to the tissue at the middle of the incision, which could weaken the abdominal wall. The space between the rectus abdominis and posterior sheath was chosen during cephalad dissection and then moved to the lateral edge of the posterior sheath to penetrate the extraperitoneal space between the lateral and middle abdomen. The dissecting plane of the lateral space was the extraperitoneal space between the peritoneum and parietal plane (Figures 2-II, 3A, 4A). We used the TEP technique for this separation. In 3 cases where there was less adhesion, we separated the extraperitoneal space along the peritoneum so that the posterior sheath remained intact (Figure 3B). In the space created by the separation, we further enlarged the extraperitoneal space within a 20- to 30-cm radius around the stoma, providing a large field for mesh placement.

**Figure 4:**
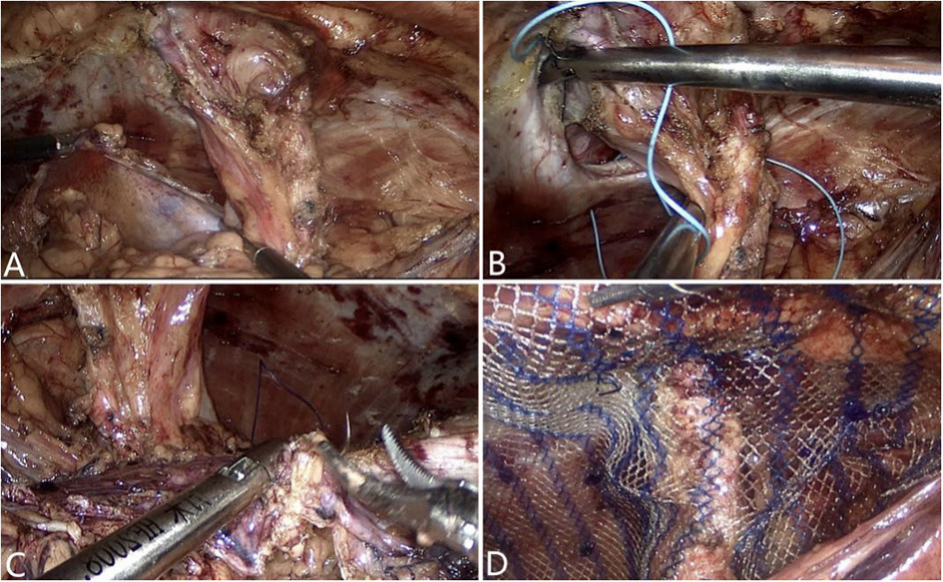
Intra operative **(A)** Dissection of the peritoneum. **(B)** Closure of the abdominal wall defect. **(C)** Closure of the peritoneum defect. **(D)** Mesh placement.

### Step 3

At the end of the procedure, the abdominal wall defect was closed with a 2-0 non-absorbable suture (Figure 4B). The peritoneum defect was secured using a 3-0 absorbable suture (Figure 4C) with the enterostomy loop positioned outside the peritoneum.

### Step 4

The polyester mesh was rolled and inserted through the 12 mm trocar. Once it reached the desired location, the mesh—which had a mean (±SD) size of 303.4 (±96.8) cm^2^–was unrolled and placed using the Sugarbaker technique so that it overlapped with the bowel, retaining it to the lateral side (Figure 4D). After the mesh was tacked and fixed in place, the entire surgical field was carefully reexamined and a negative pressure drainage tube was placed. Extrapneumoperitoneum was relieved, and the incision was closed with subcutaneous absorbable sutures.

## Results

Seven patients with a mean (±SD) age of 62.2 ± 5.7 years, mean body mass index of 28.7 ± 3.7 kg/m^2^, and average American Society of Anesthesiologists class 3 underwent hernia repair. The patient's characteristics are detailed in Table 1. None of the patients had prior parastomal hernia repair. The average parastomal hernia defect size was 3.1 ± 2.7 cm. All seven patients had end colostomies following open abdominoperineal resection for rectal cancer. Diabetes was the most frequent comorbidity, followed by hypertension and chronic obstructive pulmonary disease. Four patients were active smokers. A polyester mesh without anti-adhesion coating was used in all patients who underwent repair.

**Table 1:** Characteristics of the study population.

**Characteristic**	**Mean ± SD or *n* (%)**
Age, years	62.2 ± 5.7
**Sex**	
Male	3 (43%)
Female	4 (57%)
BMI, kg/m^2^	28.7 ± 3.7
**Comorbidities**	
Diabetes	2 (29%)
Hypertension	2 (29%)
COPD	1 (14%)
Smoking history	4 (57%)
Steroids	0 (0.0)
Hernia defect size, cm	3.1 ± 2.7
Colostomy	7 (100%)

*BMI, body mass index; COPD, chronic obstructive pulmonary disease*.

The mean size of the hernia defect was 3.1 ± 2.7 cm and the mesh size was 303.4 ± 96.8 cm^2^. The mean operative time was 195.5 ± 20.7 min. One patient developed subcutaneous emphysema (Table 2). The average follow-up time was 8.5 ± 2.7 months. Mild pain occurred in 2 patients; 3 experienced seroma formation (with no special treatment required); and there was 1 case of early intestinal obstruction (treated with conservative care). The patients were discharged an average of 4.8 ± 2.1 days post surgery. There were no stoma-related complications, mesh erosions, or intestinal obstruction in the follow-up examination (Table 3). Routine imaging performed at 3 months revealed no recurrences of hernia.

**Table 2:** Intraoperative parameters.

**Variable**	**Mean ± SD or *n* (%)**
Operative time, min	195.5 ± 20.7
Mesh size, cm^2^	303.4 ± 96.8
**Intraoperative complications**	
Subcutaneous emphysema	1 (14%)
Bleeding, ml	12.8 ± 5.5

**Table 3:** Postoperative outcomes.

**Variable**	**Mean ± SD or n (%)**
Length of hospital stay, days	4.8 ± 2.1
**Total postoperative complications**	
Pain	2 (28%)
Seroma formation	3 (42%)
Early intestinal obstruction	1 (14%)
30-Day readmission	0 (0.0)
Mortality	0 (0.0)
Recurrence	0 (0.0)

## Discussion

Parastomal hernia is a common and challenging complication following stoma creation that has a higher incidence rate than other types of incisional hernia. The main risk factors for the development of parastomal hernias are obesity, age, malignancy, inflammatory bowel disease, wound infection, steroid use, diabetes, loop ostomy, and emergency surgery (14). The laparoscopic repair of parastomal hernia with a TEP technique has rarely been reported, and there is little evidence of laparoscopic application. Laparoscopic Pauli (ePauli) repair was first reported in a peer-reviewed publication by (15) as an enhanced-view TEP repair approach that is technically feasible and has the potential advantage of avoiding intra-abdominal adhesions, but likely has a greater risk of adhesiolysis of the intestine in the parastomal hernia sack. Recently, Lambrecht et al. reported their initial experience with endoscopic preperitoneal parastomal hernia repair (ePauli repair) (16).

We successfully separated the peritoneum with a laparoscopic TEP approach (12) and repaired the parastomal hernia using a modified Sugarbaker technique. The difficulty of this approach was in the creation of an extraperitoneal space around the stoma. While dissecting, it is critical to move along the abdominal wall. We previously reported that there are several easily separated spaces on the abdominal wall (Figure 2) (12). We emphasize here that 2 types of separation can be performed in the middle abdomen—ie., between the rectus abdominis and posterior sheath, and between the posterior sheath and peritoneum (12). The peritoneum is usually extremely thin and adheres tightly to and cannot be separated from the posterior sheath. However, we demonstrated that the “space between the peritoneum and posterior sheath can be separated,” thus laying a foundation for TEP repair of parastomal hernias. In order to protect the function of the abdominal wall, the posterior sheath structure should be dissected as little as possible. In cases where adhesiolysis was difficult, we opened the peritoneum (Figure 3A); this allowed observation of the intestine within the abdominal cavity and reduced the risk of accidental injury. In cases where there was little adhesion, we moved along the extraperitoneal space (Figure 3B). There are several different approaches to entering the extraperitoneal space (16, 17) that require dissection of the transversus abdominis muscle. In contrast, with our technique it was unnecessary to release the transversus abdominis (Figure 3B); we believe that this helped to preserve the strength of the abdominal wall.

Currently, there is no uniform standard for meshes used in the Sugarbaker procedure. Meshes reported in the literature include polypropylene, expanded polytetrafluoroethylene, polyvinylidene difluoride, polyester, and biological meshes (1). In our cases, we used a polyester mesh without anti-adhesion coating. Mesh placement outside the peritoneum effectively avoids intestinal adhesion incidents and allows the use of ordinary mesh, thus reducing costs. Tack is the most common choice of material for mesh fixation; we used 5-mm titanium helical tacks and the modified laparoscopic Sugarbaker procedure to reliably fix the mesh, with mild postoperative pain and a low recurrence rate. The patients were discharged an average of 4.8 ± 2.1 days after the surgery, with no postoperative complications such as pain, bleeding, or sepsis.

With advances in laparoscopic technology, many patients with rectal cancer prefer treatment by laparoscopic surgery. For patients with a parastomal hernia after laparoscopic Miles surgery due to a small surgical scar on the abdominal wall (18), it should be easy to separate the preperitoneal space; applying the TEP approach to repair the parastomal hernia is feasible. We repaired parastomal hernias with the TEP approach in 7 patients with a history of open abdominal surgery. However, there are some technical difficulties in this operation and the operative time is long.

Compared to other laparoscopic techniques, ours has the following advantages:

1) No surgical incision around the stoma, which reduces the risk of infection.
2) Fewer intra-abdominal adhesions caused by the mesh.
3) A mesh with anti-adhesion coating is not necessary, thus reducing costs.
4) For patients with previous intraperitoneal mesh repair of midline hernia and massive adhesions, the TEP Sugarbaker approach is a probable choice.
5) In some patients, space can be created without altering anatomic structures and there is no need to release the transversus abdominis, which avoids weakening the abdominal wall to some extent.

However, our technique also has the following shortcomings:

1) Technically difficult.
2) Lack of long-term follow-up data.

Subcutaneous emphysema is a potential complication of laparoscopic surgery but is more likely to occur in extraperitoneal surgery as insufflated CO_2_ can easily diffuse into surrounding tissues. High insufflation pressure increases the risk and is the most likely cause of this complication (19, 20). The rapid diffusion of CO_2_ and occurrence of subcutaneous emphysema can be attributed to the fact that the extraperitoneal space is continuous with the subcutaneous space. Subcutaneous emphysema was managed during the laparoscopic procedure by avoiding high CO_2_ insufflation pressure, applying hyperventilation, and discontinuing N_2_O, which rapidly enters the gas space containing CO_2_ and contributes to the gas volume (21). In our case series, 1 patient developed subcutaneous emphysema. Lung ultrasound was immediately performed to exclude pneumothorax. The patient recovered within 3 days after the operation without special treatment.

In a study that used the retromuscular Sugarbaker technique to repair parastomal hernia, stoma necrosis, bowel obstruction, and perforation were observed during the long-term follow-up (22). In our patients, there were no mesh-related complications up to 8.5 ± 2.7 months post surgery. One patient had early intestinal obstruction 1 month later, which is typical after a colostomy and was unlikely to be related to the procedure that we performed. We provided conservative care and the patient recovered within a few days. A laparoscopic approach through a keyhole or slit in the mesh is associated with a high recurrence rate (37%) (23). The laparoscopic repair of parastomal hernias using the modified Sugarbaker technique with a non-slit mesh and lateralization of the bowel is promising, although the clinical efficacy, recurrence rate, and late complications require confirmation in more cases with long-term follow-up.

## Conclusion

Modified Sugarbaker repair of parastomal hernia with a TEP approach is a safe and feasible procedure, even in patients with a surgical history of open rectal resection. Although long-term outcomes are unknown, this novel approach offers a new option for the surgical treatment of parastomal hernia in the future. Further studies examining long-term recurrence rates and randomized control trials are needed in order to make definitive recommendations.

## Data Availability Statement

The original contributions presented in the study are included in the article/supplementary material, further inquiries can be directed to the corresponding authors.

## Ethics Statement

The studies involving human participants were reviewed and approved by The Medical Ethics Committee of Northeast International Hospital (2019-new-01). The patients/participants provided their written informed consent to participate in this study. Written informed consent was obtained from the individual(s) for the publication of any potentially identifiable images or data included in this article.

## Author Contributions

MW and HJ contributed to study conception and design, data acquisition, interpretation, and drafted the manuscript. DT contributed to data analysis, interpretation, and drafted the manuscript. XC and CM contributed to data acquisition and interpretation. All authors critically revised and approved the final version.

## Funding

This study was supported by The Scientific Research Projects for Higher Education in Inner Mongolia Autonomous Region (grant no. NJZZ21027), Support Plan for the Innovation and Entrepreneurship Initiation Plan for Overseas Students in Inner Mongolia Autonomous Region (grant no. MOHRSS2020122), and Doctoral Start-up Fund of the Affiliated Hospital of Inner Mongolia University for the Nationalities (grant no. MDFY2020001).

## Conflict of Interest

The authors declare that the research was conducted in the absence of any commercial or financial relationships that could be construed as a potential conflict of interest.

## Publisher's Note

All claims expressed in this article are solely those of the authors and do not necessarily represent those of their affiliated organizations, or those of the publisher, the editors and the reviewers. Any product that may be evaluated in this article, or claim that may be made by its manufacturer, is not guaranteed or endorsed by the publisher.
